# A qualitative study of developing beliefs about health, illness and healthcare in migrant African women with gestational diabetes living in Sweden

**DOI:** 10.1186/s12905-018-0518-z

**Published:** 2018-02-05

**Authors:** Katarina Hjelm, Karin Bard, Jan Apelqvist

**Affiliations:** 1 Department of Public Health and Caring Sciences, University of Uppsala, S-751 22 Uppsala, Sweden; 2 Department of Endocrinology, Malmö University Hospital, University of Lund, Lund, Sweden; 3 Department of Social and Welfare Studies, University of Linköping, Campus Norrköping, S-601 74 Norrköping, Sweden

**Keywords:** Beliefs about health/illness/healthcare, Gestational diabetes mellitus, Migrants/Africa, Prospective study, Self-care, Semi-structured interviews

## Abstract

**Background:**

Gestational diabetes (GDM) is associated with health risks for both mother and child, and is particularly relevant to migrant women and women of African origin. With today’s extensive global migration, contact with the new society and health system confronts the migrant’s culture of origin with the culture of the host country. The question is whether immigrants’ patterns of beliefs about health, illness, and health-related behaviour change over time, as no previous studies have been found on this topic. The purpose was to explore development over time, during and after pregnancy, of beliefs about health, illness and healthcare in migrant women with GDM born in Africa living in Sweden, and study the influence on self-care and care seeking.

**Methods:**

Qualitative prospective study. Semi-structured interviews, with 9 women (23–40 years), on three different occasions: during pregnancy (gestational weeks 34–38), and 3 and 14 months after delivery managed at an in-hospital diabetes specialist clinic in Sweden.

**Results:**

Beliefs were rather stable over time and mainly related to individual and social factors. GDM was perceived as a transient condition as health professionals had informed about it, which made them calm. None, except one, expressed worries about relapse and the health of the baby. Instead women worried about being unable to live an ordinary life and being bound to lifestyle changes, particularly diet, developing diabetes and needing insulin injections. Over time knowledge of appropriate diet improved, although no advice was experienced given by the clinic after delivery. The healthcare model was perceived as well functioning with easy access but regular follow-ups were requested as many (decreasing over time) were unsure whether they still had GDM and lacked information about GDM and diet. During pregnancy information was also requested about the healthcare system before/after delivery.

**Conclusions:**

Beliefs changed to a limited extent prospectively, indicated low risk awareness, limited knowledge of GDM, irrelevant worries about future health, and being unable to live a normal life, associated with problematic lifestyle changes. Beliefs about the seriousness of GDM in health professionals influenced patients’ beliefs and health-related behaviour. The healthcare organisation urgently needs to be improved to deliver appropriate and timely information through competent staff.

**Electronic supplementary material:**

The online version of this article (10.1186/s12905-018-0518-z) contains supplementary material, which is available to authorized users.

## Introduction

According to the International Organization of Migration (IOM), there is currently extensive global migration, leading to multicultural societies, not least in Europe. Differences have been found in beliefs about health and illness in women with gestational diabetes mellitus (GDM) born in Africa and Sweden [1]. Migrant women from Africa did not know the cause of GDM, had a passive self-care attitude, many reported being informed by staff that GDM was transient, had limited knowledge about GDM and the body and stated more pregnancy-related problems for which they received no treatment, in contrast to Swedes who had a higher risk awareness and thus feared developing type 2 diabetes and worried about the baby’s health, used more medications against pregnancy-related complications and were more often on sick-leave from work [1]. In the process of acculturation to life in the new society the migrant is confronted with the new culture and the healthcare system in the host country [2], which may lead to changed individual beliefs about health, illness, and health-related behaviour, e.g. in terms of self-care measures and care seeking behaviour. It is interesting to follow whether the pattern of beliefs change over time. No studies have been found in the literature review examining the development of beliefs about health, illness and healthcare over time in migrant African women, which is the aim of this study.

## Background

GDM is associated with health risks for both mother and child [3] which are further increased in migrant women [4] and women of African origin [4, 5]. There is a potential risk of developing glucose intolerance and type 2 diabetes in the future for the mother [6]. The lifetime risk of developing type 2 diabetes is 7–12 times greater in women with GDM in a previous pregnancy [7]. For the child there is an increased risk of congenital abnormalities, perinatal mortality [3, 8], preterm birth and macrosomia because of maternal hyperglycaemia [9, 10], and with a further increased risk for migrant women [8, 11, 12]. Normalised glycaemic control during pregnancy by well-managed GDM can reduce the risk for the child [6, 13–15]. Knowledge and self-care measures such as diet, exercise, medications and self-monitoring of blood glucose are essential, but demanding, measures to normalise blood glucose [6, 14]. Pregnancy has been described as a period of transition and transformation to motherhood for the woman [16], implying physical, mental and social changes [17] which are further stressed in women with GDM. GDM is associated with lifestyle changes and emotional reactions due to the treatment, and the condition is perceived as potentially life-threatening because of the diagnosis and its treatment [18]. Information about the diagnosis may be distressing to the woman [19, 20]. Migrant women are exposed to additional demands through the acculturation process, to which they also have to adapt [2]. For healthcare providers pregnancy is an opportunity to change lifestyle patterns into healthier habits for the individual and society, through education about the future risk of developing type 2 diabetes mellitus and measures to prevent it [6, 13, 14]. Type 2 diabetes mellitus can be prevented or the incidence delayed by weight reduction, regular exercise and healthy dietary habits [6, 15, 21].

Previous studies focusing on beliefs about health and illness in persons with diabetes mellitus of different origin have shown that North Africans mentioned stress or fate as a cause of diabetes mellitus [22], in contrast to Europeans who mentioned medical factors such as heredity or obesity [22–25]. A similar fatalistic view of the disease and an external locus of control, through fate or the influence of supernatural factors (e.g. God or Allah), were described by migrants with diabetes mellitus from Somalia [25], former Yugoslavia or the Middle East [23–25] and Yemen-Arabs in the USA [26].

Health was described as freedom from disease, or from a pathogenic perspective, by Swedes, persons from Former Yugoslavia, and Middle Easterners, although different self-care behaviours were described in the three latter. Swedes described active self-care behaviour with a controlled and healthy lifestyle; persons from Former Yugoslavia held a passive attitude to self-care and tried to enjoy life by deviations from advice received, particularly about diet; Middle Easterners exemplified an active information-seeking behaviour, with a low threshold for seeking healthcare, and wished to adapt to the diabetic disease [23–25]. Thus, migrants with diabetes mellitus perceived the disease as less serious, described limited knowledge about the body and the disease, and lower degree of self-efficacy. A previous survey on attitudes to GDM in a multiethnic population found women with a non-Caucasian background at higher risk of poorer self-management due to poorer health literacy and poorer comprehension of their condition [27].

The migrant population in Sweden is a mixture of persons from more than 200 different countries, with African migrants being the second biggest non-European migrant group following those originating from the Middle East [28]. Most come from Somalia and Ethiopia and have mainly fled from war and persecution during the 1990s and are thus asylum-seeking refugees or have family ties as the main reason for immigrating [28, 29].

### Aim

The aim of the study was to explore the development over time, during and after pregnancy, of beliefs about health, illness and healthcare in migrant women with GDM born in Africa living in Sweden, and also to study the influence on health-related behaviour, including self-care and care seeking.

### Management of gestational diabetes

Screening for GDM was done by a midwife at a healthcare centre in the 28th or in the 12th gestational week in the case of heredity of diabetes mellitus or previous GDM. If tested positive the woman was referred to an out-patient specialist diabetes clinic. Staff in the clinic were organised in a diabetes care team working with all kinds of diabetes. Management during pregnancy and after delivery is described in more detail in a previous study [30]. According to a national child health programme [31] regular visits to the child healthcare clinic were made.

### Theoretical framework

The theoretical framework guiding the study is previously described [1, 25]. Health-related behaviour is explained by the Health Belief Model (HBM; [32]) where perceived threat from a disease guides the actions, based on the lay theory model of illness causation (to be found in the individual, natural, social and/or supernatural sphere) giving the explanation of the disease [33] and determining from which sector in society healthcare should be sought, either the popular sector (family, friends or relatives), or the professional or folk sector from health professionals or folk healers (sacred or secular), as described in the help-seeking behaviours model [34]. Perceived locus of control then influences the degree of activity in health-related behaviours [35]. Perceived personal control over the environment promotes actions, in contrast to feeling governed by factors outside one’s own control, such as fate, luck, and chance, leading to less likelihood of engaging in self-management, internal versus external locus of control.

Individual beliefs are learned through socialisation with others (e.g. family, schools, healthcare etc. [36], and influence attitudes determining health-related behaviour, and are based on knowledge held by the individual [37]. Thus, beliefs about risks or risk awareness are culturally determined and based on knowledge but also depend on the trustworthiness of the information source. Intra- and interpersonal factors, institutional or organisational factors, community factors and public policy factors influence health-related behaviour and a reciprocal causation between the environment and the individual is evident, as behaviour is influenced by and influences the social environment [37].

According to the theory of convergence [38] differences in health will exist between generations as lifestyle and behaviour are not inherited. Thus, it is reasonable to assume changes over time in migrants through an acculturation process which poses new demands on the individual [2], added to those from the transition to motherhood [16]. Trying to adapt to the lifestyle and behaviour in the new country may result in stress that influences health beliefs, health behaviour and health [2].

In international studies African societies, although diverse, have been described as group-oriented with large power distance, low individualism and need for rules [39]. Being brought up under these conditions with dependent collectivism [39] where one learns to rely on and obey authorities, e.g. health professionals, might negatively influence the degree of self-efficacy and lead to less independent behaviour and low perceived ability to act on their own [40]. The degree of self-efficacy, and thus health-related behaviour, is also influenced by transitions, particularly stressful ones such as migration [41], mastery experiences, mood [40], and cultural factors [42].

## Methods

### Design

A qualitative prospective exploratory study design was chosen. Semi-structured interviews on three different occasions were used to collect data. Interviews were chosen to reach a deeper understanding of the individuals’ own perspectives when allowed to tell their stories freely [43].

### Participants

Nine women born in African countries, diagnosed with GDM, and living in Sweden were included. Inclusion criteria were age 16 years or above, and diagnosis of GDM (O 24.4 – Gestational Diabetes Mellitus; according to the International Classification of Diseases version IX 1997). A convenience sampling procedure was used, and health professionals at an in-hospital specialist diabetes clinic invited all female patients diagnosed with GDM to the study.

### Data collection

A semi-structured interview guide with open-ended questions was used for each interview, which began with standardised questions on socio-demographic and medical background data. The interview guide had been developed on results from previous studies [23, 44, 45], a review of literature focusing on pregnancy and health, and had finally been peer-reviewed by experts in management of GDM; diabetes specialist nurses, diabetologists and midwives (Additional file 1). Themes explored were beliefs about health, illness and healthcare; causes, explanations and consequences of GDM; factors of importance for health; self-care advice, self-reported adherence to advice received; and healthcare seeking.

### Procedure

Interviews were held on three occasions, in gestational weeks 34–38 and 3 and 14 months after delivery. The interview guides were pilot-tested in three women (not included in the study), and some minor changes were made (mainly wording). A female diabetes specialist nurse (first author), not involved in the studied clinic or the management of the women, led the interviews. An authorised female interpreter was used when needed (in all except two cases) and interpreting word for word (sequential interpretation technique) as recommended in Sweden [46]. The interviews were held in secluded rooms outside the diabetes clinic, lasted about 1.5 h, were audio-taped and then transcribed verbatim. The texts were coherent and as they showed no non-translated parts the quality was considered high. After the interviews and during data analysis questions concerning culture-specific phenomena were discussed with the interpreters. This was done to compensate for second-order interpretations as only a native can make first-order interpretations [47].

### Ethical considerations

The study was carried out in accordance with the Helsinki Declaration, with written informed consent, and had been approved by the Ethics Committee of Lund University. During the interviews, the interviewer was observant of signs of distress in the respondents, who could, if judged necessary, be referred for counselling to a social worker or psychologist, but no problems were identified.

### Data analysis

Data were simultaneously collected and analysed and until no new information was added when analysing data from new respondents [43]. Content analysis was performed according to a method described by Krippendorff [48]. The aim in the analysis was to be open to as much variation in the material as possible, and to search for patterns, themes and contradictions by comparing the informants’ statements at each interview, and also making comparisons of content between the three different interviews so as follow development over time. After each interview, the tapes were listened through and notes were taken about themes, emerging ideas and general findings. First the text was read through as a whole, then each line of the text was reviewed, topics were identified, and finally the material was extracted and condensed into categories of content [48]. Categories were then developed inductively and titled as close to the text as possible. Categories from theoretical models were also brought into the material [43]. The lay theory of illness causation [33] and the model of help-seeking behaviours [34] served as main analytical categories (for details see [23–25]).

In order to increase the validity of the findings a diabetes specialist nurse and a general nurse (first and second author) were involved in the analysis of data [48, 49]. The first author checked the content of categories developed and comparisons with the second author showed high agreement. Quotations were used to illuminate the results and verify the categorisation.

## Results

The respondents’ age range was 23–40 years (Md 31 years) and they were born in countries in Africa (Algeria *n* = 1, Ethiopia n = 1, Gambia n = 1, Morocco *n* = 2, Somalia *n* = 4). All participants were refugees, five because of family ties. The median time of residence in Sweden was 6 years (range 1–15) and all had received their diagnoses of GDM in Sweden, four of them diagnosed during a previous pregnancy. The majority were parous (*n* = 8) and had been treated with insulin during pregnancy (*n* = 5). Educational level varied, with about half with limited education (< 6 yrs. *n* = 1, < 9 yrs. *n* = 4, upper secondary school *n* = 3, university > 2 yrs. *n* = 1); most were students (*n* = 6) and only two had a job.

### Beliefs about illness

In talking about the development of feelings over time^1^ when looking back on the time when the woman had been informed about the diagnosis of GDM, most described being sad and worried, one had lost her appetite, another searched for information and some said they didn’t think it was particularly serious as they didn’t know about the disease (Table 1). They calmed down after information from health professionals and this persisted at both interviews, 3 and 14 months, after delivery; only one person talked about the risk of relapse at both interviews.

1: I felt worried and sad, I went home to cry…that was before I met the physician who said ‘it is easy to prevent and it will possibly disappear after delivery’ (R33, antenatal).

1: In the beginning I didn’t know how serious this disease was…I didn’t perceive it as particularly serious (A6, antenatal).

After delivery most said they thought that the GDM had gone. At the last interview one person held supernatural beliefs and said that 3: ‘God decides…but it is important to manage your own health’ (A6, 14 months).

Most respondents claimed initially during pregnancy that GDM is temporary and would disappear after delivery, further emphasised by some who said they had been informed by the physician that it is a transient condition. Others said they didn’t know how long it would last and some mentioned being informed about the transience of the disease by the physician during pregnancy. After delivery fewer said that they didn’t know but that they had been informed that the disease would disappear after delivery, while others said they didn’t have it any longer but also expressed uncertainty about whether they had been cured or not. Fourteen months after delivery most claimed they didn’t have GDM any longer but expressed uncertainty, and one mentioned the risk of relapse while a couple said that God decides (supernatural factors).

1: Actually I don’t know…the second test showed normal values…don’t know whether it has disappeared (A10, antenatal).

2: I am still unsure if I have sugar…but she said (physician) they will test next year again (A7, 3 months)

3: I am free from problems with diabetes right now…the midwife has reminded me that it can happen that it returns later in life (A5, 14 months)

Discussions of the consequences of GDM for health showed that during pregnancy most expressed no influence or they hoped it would not affect health negatively. Three months after delivery no problems were mentioned except a few who said they had no time for themselves and a few that it was in the hand of God (supernatural influence). Finally, most reported no problems 14 months after delivery and a few spoke of worries about health.

A major problem expressed throughout the interviews, mostly emphasised during the second interview, was difficulty with change of lifestyle, particularly diet. Physical problems related to pregnancy, e.g. headache, nausea and vomiting, were described in the first interview, stress and worries in interviews 1 and 3. Over time a decreased number of respondents claimed that nothing had a negative influence on health. Factors exemplified as harmful were not managing oneself, problems with weight, relations or language.

2: I have started to reduce these sweet things, fat, and started eating brown bread instead of white. Yes of course it was difficult…difficult to go into the shop and look for these light products (A3, 3 months).

1: Some meals you have to take at the right time, …it is the diet also…it’s very difficult to drink without sugar or with a special kind of sugar…one problem is that you can’t live as usual, you have to control yourself, refrain from all kind of sweets…I am very fond of biscuits…can’t take what I want… (SFW, antenatal)

3: She is thinking a lot about whether she will get real diabetes in the future and that she can’t eat as much as she wants (A6, 14 months)

Most respondents expressed worries about not being able to live a normal life, instead being bound to lifestyle changes (not eating sweet things) and diabetes, about relapse and the need for insulin injections, although more pronounced in the first and last interviews. Two women discussed fears for the health of the child in the first interview. In only one case, at the last interview, one person talked about fears of diabetes-related complications.

No factors were identified in any of the interviews that were perceived as negatively affecting health, with the exception of problems with weight mentioned by two women in the first and one woman in the last interview.

As regards worries about health and future health, this was discussed to a limited extent, and then it was mainly focused on the woman’s own health in general, particularly in the first and last interviews. The women were hoping for good health and a few exceptions mentioned worries about the child in the first postpartum interview. In this and the last interview supernatural factors and reliance on the will of Good were also mentioned. On these occasions the importance of being careful and managing one’s own health in general was also brought up.

1: I don’t think it will affect the baby…but I don’t think so and I hope it doesn’t. But for my own sake it might return when I start ageing. (57, antenatal)

3: I think you have to fight so that you don’t get this disease again. You can get it as an inheritance, but I hope I will not get it in the future. (A4, 14 months)

3: I can’t decide about the future. It’s God that decides it. (A3, 14 months)

**Table 1:** Description of development of beliefs about illness in migrant women with gestational diabetes mellitus (GDM) born in African countries living in Sweden

Variable	Gestational week 34–38	3 months after delivery	12 months after delivery
Emotions related to being informed about the diagnosis of GDM	Sad, worried Not perceived as serious One lost appetite One searched for information Calmed down after information from hc staff	Calm after information from hc staff Risk of relapse stated by one	Calm after information from hc staff Risk of relapse stated by one
Beliefs about duration of GDM	During pregnancy	It is gone but unsure whether cured, might come back	Most said it has gone but unsure whether cured
	The physician has said it is transient, will disappear after delivery	Fewer said they didn’t know but had been informed by health professionals that it would disappear.	Risk of relapse stated by one ‘God decides’, important to manage your health^b^
Consequences of GDM for health	No problems Hope it will not affect/influence health negatively	Fewer claimed no problems No time for oneself The will of God^b^	Even fewer claimed no problems Worried about health
Problems related to GDM Worries related to having been diagnosed GDM	Difficulties changing lifestyle^a^ Physical health problems; headache, nausea, vomiting^a^ Stress and worries related to GDM^a^ Fear and worry about - not being able to live a normal life, being bound to life-style changes (not eating sweet things) - of having diabetes, relapse of GDM, insulin injections Fear for the health of the child mentioned by two women	Difficulties changing lifestyle, particularly diet emphasised here^a^ Compared to during pregnancy less pronounced fear and worries about - not being able to live a normal life, being bound to life-style changes (not eating sweet things) - having diabetes, relapse of GDM, insulin injections	Difficulties changing lifestyle^a^ Stress and worries related to lifestyle changes^a^ Fear and worry about - not being able to live a normal life, being bound to life-style changes (not eating sweet things) - of having diabetes, relapse of GDM, insulin injections of having diabetes-related complications mentioned by one woman
Beliefs about future health	Hope for good health	Hope for good health Worries about future health of the child and herself God decides^b^	Hope for good health God decides^b^

As main analytical categories in data analysis the following have been used, when appropriate, according to the lay theory model of illness causation by Helman [33]: ^a^Individual factors; ^b^Supernatural factors

### Beliefs about health

Throughout the interviews the core description of health was freedom from disease but mental well-being was also important (Table 2). During pregnancy it was mainly described as factors related to the individual, such as lifestyle (diet and exercise). Social factors such as the importance of health in order to be able to fulfil the role of mother were discussed to the same extent in all interviews, as well as the influence of God and the supernatural world, which was mentioned by a few.

2: Health is when you are and feel completely healthy and without sugar…without all diseases. (A4, 14 months)

Lifestyle factors such as diet and exercise (individual factors) were mentioned at all three interviews as factors of importance for health. However, this was less pronounced in the second interview where some also stated they did ‘nothing’. In the first interview during pregnancy meals were also discussed, as well as a combination of individual and social factors focusing on following advice concerning diet, meals, controls and medications.

1: to eat in the right way, exercise or such things…the food they have described to me and avoid all these other things that I normally eat…Mostly with these sugary things…(R, antenatal)

As factors detrimental to health, ‘nothing’ was reported by most respondents at all three interviews. Matters of ‘weight’ and ‘overweight’ were brought up in the first and last interview by a few persons.

Individual factors such as diet and exercise were mentioned as important for maintaining health and preventing complications related to GDM in all interviews, although less and particularly concerning exercise in the second interview 3 months after delivery. During this interview weight reduction was also mentioned.

Home remedies or alternative medicine measures were not used by anyone in the interviews. Nor was there any change in beliefs about the importance of religion for health. Praying was considered important, as most are devout Muslims and say that ‘religion is important for health’. As regards celebrations of feasts and traditions some said it had positive effects on health while others that it has a negative influence. However, after delivery many claimed at both interviews that celebrations are important for health as they make people come together and give an increased feeling of well-being by socialising with others. With a few exceptions contact with and information about self-help groups concerning diabetes or families are almost absent.

2: It makes you more happy to meet relatives (A8, 3 months)

3: …I’m happy though…we go and visit each other, we come together and we wear nice dresses (A5, 14 months).

Over time what was considered healthy diet changed from limited descriptions of reduction of sugar and fibre-rich food during pregnancy to more detailed explanations also including reduction of fat and salt a year after delivery (first and third interview). However, regular meals and the plate model were only discussed during pregnancy (first interview).

1: …vegetables and eating regularly…not sweets or chocolate (R 32, antenatal)

3: …important to eat good food….if you eat much fat and sugar it can harm. Or much salt (A4, 14 months)

Most respondents considered a diabetic diet more expensive than ordinary food, and reported difficulties here. Another small group considered personal finances unimportant for their health and claimed to have a good economy.

2: …diabetes food is very expensive…You have to buy it. It is health that is in focus. It happens that you don’t have money to buy food (A3, 3 months).

2: Your economy is of no importance. Health is one thing and economy another (A4, 3 months).

Exercise in terms of ‘taking walks’ (1: R 33) was considered appropriate for health in all interviews.

As regards self-monitoring of blood glucose, most respondents know when to use this during pregnancy but with varying frequency (4 times daily to every second day) throughout the interviews. However, the number of respondents stating that they did not use SMBGs increased over time after delivery. Those who had received advice about controls also increased over time, but the majority in interview 3 had not received any advice.

1: …four times a day…I have to follow the doctor’s advice ((R55= A7, antenatal)

1: …to test every other day (R 56=, A4, antenatal) I: …Before and after meal?

2: sometimes they said daily, others every other day.. it differs from time to time (A5, 3months).

2: At the delivery ward twice a day…then I have given back the blood glucose machine (A4, 3 months)

3: I have not got any advice…nothing…They said they would call me (A:1, 14 months)

**Table 2:** Description of development of beliefs about health in migrant women with gestational diabetes born in African countries living in Sweden

Variable	Gestational week 34–38	3 months after delivery	12 months after delivery
Health described as	Freedom from disease^a^ Mental well-being^a^ To be healthy to be able to take care of the child^a, b^	Freedom from disease^a^ Mental well-being^a^ To be healthy to be able to take care of the child^a, b^	Freedom from disease Mental well-being To be healthy to be able to take care of the child ^a, b^
	God decides^c^	God decides^c^	God decides^c^
Factors of importance for health	Manage diet and meals^a^ Exercise^a^ Follow advice concerning diet, meal times, controls, medications^a, b^	Appropriate diet^a^ Exercise^a^ Nothing particular	Appropriate diet^a^ Exercise^a^
Factors with negative impact on health	Nothing, except two stating weight^a^	Nothing	Nothing, except one stating weight^a^
Maintenance of health and prevention of complications	Diet/exercise^a^	Diet/exercise but to a lesser extent and particularly concerning exercise^a^ Weight reduction^a, b^	Diet/exercise^a^
Use of alternative medicine/home remedies to improve health Religion of importance for health	Not used Religion important for health for all except one^c^	Not used Religion important for health for all except one^c^	Not used Religion important for health for all except one^c^
Celebration of feasts/traditions of importance for health	Most told of positive influence on health and some that it had negative influence^a^	Many told of positive influence by increased well-being by meeting others^b^	Many told of positive influence by increased well-being by meeting others^b^
Beliefs about appropriate food	Rich in fibres Reduction of sugar Regular meals The plate model	High fibre, sugar reduction, low fat diet	High-fibre, sugar reduction, low-fat and salt-reduced diet
Beliefs about appropriate exercise	Taking walks is good	Taking walks is good	Taking walks is good
Influence of economy on health	Most considered DM more expensive than ordinary food affecting health A few said economy had no influence on health as they had good economy	Most considered DM more expensive than ordinary food affecting health A few said economy had no influence on health as they had good economy	Most considered DM more expensive than ordinary food affecting health A few said economy had no influence on health as they had good economy

As main analytical categories in data analysis the following have been used, when appropriate, according to the lay theory model of illness causation by Helman [33]: ^a^Individual factors; ^b^Social factors; ^c^Supernatural factors

### Beliefs about healthcare

Access to healthcare in terms of diabetes care and healthcare in general was described by most as unproblematic and easy throughout pregnancy and after delivery, with a few exceptions mentioning the limited time for consultations, but these were only found in the first interview during pregnancy (Table 3). However, there was also a group of patients who didn’t know as they were called for regular check-ups.

1: They just had a short time so there was no chance to tell how you feel (R49, antenatal)

1: I haven’t had any problems. If I want to see a particular person I get an appointment when I call…I get help and controls (R 55, antenatal).

Communication and contact with healthcare staff was described on all three occasions without any great problems except limited time for consultations, as mentioned by a few in the first interview during pregnancy. Most speak Swedish and thus use interpreters to a limited extent. Both professionals and relatives (husband) were used as interpreters if needed.

There seemed to be a tendency, from mostly following advice received during pregnancy to less compliance with advice over time after delivery, when more said ‘almost follow’. 3: yes some (I follow)…some I had to follow during pregnancy and some you can follow after pregnancy…low-fat food, keyhole products, sugar-free lemonade..(A:2).

Health professionals were perceived as an important source of information throughout the interviews while their importance for ‘taking care of’ was stated more frequently and 3 and 14 months after delivery ‘don’t know’ was mentioned less often. In the last interview one person said ‘nothing’ as she said that you have to take your own responsibility.

A good, or ideal, nurse or doctor was described as one having a positive attitude to the patient by being ‘calm and a good listener’ (2: A4) throughout the interviews and ‘giving proper information…and having knowledge’ (3:A5). The respondents expected them to provide help and support.

The present healthcare model was perceived as functioning well throughout the interviews and the respondents felt they got the help they needed. However, in the interview during pregnancy there were requests for more information about healthcare before and after pregnancy. With increasing frequency over time there was a desire for more support and regular check-ups, particularly emphasised in the third interview.

1: I think it is good as it is…but I didn’t know how it (the healthcare organisation) worked before (pregnancy) if I had known that before (R56, antenatal)

3: More contact after delivery. At least three times a year, test my blood. Interviewer: …talk about diet etc.? Yes, actually (A:1, 14 months).

**Table 3:** Description of development of beliefs about health care in migrant women with gestational diabetes mellitus (GDM) born in African Countries living in Sweden

Variable	Gestational week 34–38	3 months after delivery	12 months after delivery
Healthcare access	Easy and unproblematic Don’t know, regularly called for check-ups Limited time for consultations	Easy and unproblematic Don’t know, regularly called for check-ups	Easy and unproblematic Don’t know, regularly called for check-ups
Attitude to advice given from the clinic	Advice followed	Advice sometimes or almost followed	Advice almost followed
Communication with health professionals’	Good and mostly unproblematic Limited time for consultations Most speak Swedish, all except one satisfied with interpreters Husband interprets	Good and mostly unproblematic Most speak Swedish, all except one satisfied with interpreters Husband interprets	Good and mostly unproblematic Most speak Swedish
Advice given about diet from the clinic	Diet rich in fibre, sugar free and fat-reduced	Most have not got any information about diet, some describe the same as during pregnancy One advised reducing weight, another stop eating sweets, chips	Most have not got any information about diet, some describe ‘diabetes diet’, another ‘light products and vegetables.
Advice given about exercise from the clinic	All except two informed about the importance to take walks	One group advised to take walks Another group got no advice	Most have got advice about the importance of exercise
Advice given about SMBG from the clinic	Varying; 4 times/day 4 times every second day	None stated by most, a few testing	None stated by all except two (2 times/week or sometimes)
Beliefs about the present healthcare model	Well-functioning	Well-functioning	Well-functioning Lack of/desire for regular and more frequent controls and information about the disease
	Need of more information about health care before and after pregnancy	More information and advice	
Expectations on health professionals: the good/ideal nurse or physician	Calm and a good listener Give proper information and be competent (having knowledge) Give help and support	Calm and a good listener Give proper information and be competent (having knowledge?) Give help and support	Calm and a good listener Give proper information and be competent (having knowledge?) Give help and support

## Discussion

The main finding was that beliefs were rather stable over time. Most noticeable is that GDM was not perceived as particularly serious but as a transient condition, further amplified by health professionals’ information that had made them calm down. None, except one, expressed worries about relapse and the health of the baby. Instead they worried about being bound to stressful lifestyle changes, particularly diet. Over time the knowledge of appropriate diet improved although no advice was perceived to be given after delivery. The healthcare model was described as well functioning but regular follow-ups were desired as many (decreasing over time) were unsure if the disease had disappeared and lacked information about GDM and diet. During pregnancy information was also requested about the health system.

This study is unique as it explores the development over time of beliefs about health, illness and healthcare in migrant African women with GDM during and after pregnancy (3 and 14 months). Thus, comparisons with previous studies can only be partial.

Risk awareness in women with GDM in this study, in contrast to a U-shaped relapse (no worries and lifestyle held before diagnosis 3 months after delivery) in women from the Middle East in a previous study [30], was rather unchanged over time although low, indicating limited knowledge about the condition and the risk for future health. The difference might be related to socio-demographic factors such as educational level, beliefs, attitudes and previous experiences of diabetes from the home country and treatment in Sweden, basic knowledge about the body and diseases [32, 37], perceptions of pregnancy (normal state not requiring medical attention or not) [50], the role of the mother and the connection to the child [51], time of residence in Sweden (longer here), migration experience or background (more refugees here) that influence beliefs and thus health-related behaviour [37]. Religious beliefs also exert influence [24], expressed in different ways but less pronounced among women born in African countries in this study population compared to e.g. women from the Middle East. However, other main influencing factors underpinning beliefs are perceived seriousness of the disease among healthcare staff [52] and the influence of the healthcare organisation [53]. Women in this investigation stated being informed about the transience of the disease, as previously reported in migrant women from the Middle East [25] but to a higher extent. This r educed the stress they perceived during and after pregnancy. The difference may be that the more active information-seeking behaviour previously found in Middle Eastern women [24, 25, 30] might have compensated for lack of advice from health professionals concerning management of GDM after delivery and onwards, in contrast to the studied group who expressed wishes to get help and support from staff, indicating a more passive attitude. Beliefs about health and thus health-related behaviour are at least affected by individual, or intrapersonal factors, organisational or institutional factors and public policy and community factors [37] which need to be considered. A reciprocal relation exists between individuals and their environment, and behaviour is influenced by and also influences the social environment.

Giving appropriate advice and particularly communication about health risks is a balancing act between preventing unnecessary worries and increasing the person’s risk perception by delivering facts and supporting advice [37, 54, 55]. Many women in this study expressed stress in all interviews, as previously described in a literature review of women’s experiences of GDM [56]. The stress was particularly related to adjustment of dietary habits, perceived as a problem, and more tailored education might decrease this dietary management stress described in an empirical study [57].

Women in this study expressed incorrect beliefs about the risks of GDM for future health, with the main concern being inability to live an ordinary life because of lifestyle changes, particularly diet. Previous studies of Somalian immigrants with diabetes living in Sweden [58] showed that many felt a lack of freedom due to strict everyday management, uncertainty as to what traditional food to eat, and difficulty giving up traditional habits. However, advice about the general principles of a healthy diet after delivery, aimed at preventing type 2 diabetes, should not cause these worries. This indicates lack of knowledge related to insufficient information about diet after delivery and no clear explanations of the difference in dietary habits compared to what is important during pregnancy and being on insulin treatment (as most were), where strict meal planning and stricter dietary advice is given to achieve good glycaemic control. Further, as food can be a means of constructing ethnic identity in the acculturation process in the new country [59] it is important to assess dietary habits and offer culturally adapted food advice to develop healthy habits. The cultural distance, defined as dissimilarities in cultural values, social structure (e.g. family), religion and living standard [60], between persons of African origin and Swedes seems big and might exert an influence. A previous systematic review found a need for lifestyle change support in women with GDM [61].

Despite the claimed lack of information about diet from healthcare the respondents’ knowledge about the content of appropriate diet had developed over time. Thus, they must either have sought more information on their own or gained from discussions during the interviews and a possible intervention effect [37].

As regards beliefs about factors of importance for health, the interest in dietary habits and exercise (individual factors), along with uncertainty among some as to whether GDM was transient and not present any longer, indicated a perceived internal locus of control and the possibility to influence health [35] and thus degree of self-efficacy and health-related behaviour [40].

Women in this study described health as freedom from disease and well-being, as previously found in Somalians with diabetes [62] and in Middle Easterners with GDM living in Sweden [30]. However, the Somalians also held a fatalistic view [62] related to supernatural beliefs [33]. The influence of supernatural factors was discussed here to a limited extent even though the majority reported being devout Muslims. This indicates that beliefs about health and illness are individual and need to be assessed for individual care planning. This is further supported as beliefs mainly were related to individual and social factors (lifestyle and social relations) instead of focusing on supernatural and social factors as proposed among non-westerners, although this is not empirically tested [33]. Thus, changes in beliefs over time, with one exception [30] not previously studied, are related to situational and cultural factors.

Access to healthcare was perceived as easy and communication with healthcare staff as unproblematic, in contrast to findings from previous studies of migrant women with GDM from the Middle East [25, 30]. This is possibly because most of the African-born women were Swedish-speaking and few needed help from interpreters or if so, they were pleased with those offered, in contrast to Middle Eastern women.

The healthcare model studied was perceived as well functioning but respondents requested regular follow-ups as many were unsure about their health status and lacked information about GDM and particularly diet both during pregnancy and after delivery. As in a previous pilot study, many complained about being left with doubts as information about GDM had been limited [63]. Management stress [56] and dietary management stress [57] developed additionally to the acculturation stress that migrant women face when adapting to life in a new country [2]. Development of appropriate coping strategies will then be prevented and might negatively influence the sense of self-efficacy determining the degree of participation in self-management [40] and thus, affect health negatively.

The expectations on health professionals also underline the need for information and thus, as shown in a review of help-seeking behaviour in women with GDM in the postpartum period, there is a need for lifestyle change support [61] from diagnosis of GDM throughout pregnancy and also after delivery. During pregnancy, and up to a year after delivery, the content of patient education needs to be adapted to the need, as health beliefs are situational and culturally determined [30]. Building awareness of the seriousness of the consequences of GDM for both mother and child is needed during pregnancy, focusing on enabling self-care [15]. After delivery regular follow-ups and lifestyle change support should be emphasised, particularly diet/nutritional counselling, to develop healthy habits and weight reduction to prevent type 2 diabetes.

In the studied healthcare model, the follow-up 8 weeks after delivery was focused on family planning and control of health status and not particularly on management of GDM and not delivered by health professionals with a particular role of teaching patients or trained in patient education, which is a limitation that needs to be changed. Further, developing provider awareness through professional education is needed [15] to increase knowledge of the seriousness of GDM.

As beliefs about health and illness influence health-related behaviour, including help-seeking behaviour [24, 25, 30], it is also important to include requested information during pregnancy about the health system in the new country. Pregnant women, and particularly migrant women with GDM, are vulnerable, and this may be further increased as they come from countries with different health systems and have limited knowledge and different expectations than what might be available in the new country [33, 34]. Migrant women of African origin need help in the transition into motherhood, to focus not only on their own health but also on the child’s health [16] through appropriate information delivered repeatedly during pregnancy and after delivery.

Pregnancy constitutes an opportunity for health professionals to change lifestyle patterns to healthy habits for both the individual and society [6, 13, 14], to provide health education and activities to prevent type 2 diabetes [64]. The respondents’ desires for more information and regular follow-ups after delivery, need to be met. Of particular importance is the follow-up 6 weeks after delivery, which should focus on discussions about GDM to develop increased awareness of the condition as a risk marker for development of Type 2 diabetes and the possibility to prevent it. The message of a ‘healthy diet’ and regular exercise to prevent overweight should be emphasised [21]. Important is also the influence of health professionals’ beliefs about the seriousness of the disease, which needs to be developed [52]. Health professionals thus need education, focused on the importance of assessing and planning educational activities based on the individuals’ own beliefs and delivering true and non-scaring information to develop realistic beliefs and positive health behaviours.

### Study limitations and strengths

The sample included migrant women from different African countries, and differences in language, religious beliefs, experiences of different health systems, migration experiences/background, time of residence in Sweden, degree of acculturation and integration cannot be excluded. However, they share the fact that they come from the same continent dominated by Islam and most said they were devout Muslims, all reported being refugees, and now living in the same country, having brought habits, traditions, and lifestyles from their home countries and being exposed to new ones in the host country. Educational level varied, with about half low educated. Beliefs about health and illness and health-related behaviour could be influenced by educational level [32] but no dissimilarities were found in within-group analysis. All the factors mentioned might influence beliefs and help-seeking behaviour, and not being able to establish the determinant one might be seen as a limitation. However, the studied population is to be considered as representing the population of migrant women from Africa visiting a Swedish maternity clinic.

## Conclusions

In conclusion, beliefs about health, illness and healthcare changed to a limited extent through the studied period, from during pregnancy and 14 months after delivery, and indicated low risk awareness and limited knowledge about GDM and irrelevant concerns about future health and being unable to live a normal life and instead being bound to problematic lifestyle changes due to lack of information. Beliefs about the seriousness of GDM in health professionals and the healthcare organisation influenced the development of patients’ beliefs. Thus, it is important to be aware that there is a reciprocal relationship between the individual and the environment and that behaviour affects and is affected by the social environment. The healthcare organisation needs to be developed to deliver appropriate and timely information through staff with skills and education specific to patient education throughout the trajectory of GDM and subsequent health issues.

## Additional file

Additional file 1: Interview guide. (PDF 240 kb)

## Abbreviation

GDM: Gestational diabetes mellitus

## Footnotes

1 Numbers 1, 2, 3 in the ‘Findings’ section refer to interview number 1 during pregnancy (antenatal), number 2 three months after delivery, and number 3 fourteen months after delivery.
