# Supplementary material for: A qualitative study of developing beliefs about health, illness and healthcare in migrant African women with gestational diabetes living in Sweden
Source: BMC Womens Health. 2018 Feb 5;18:34. doi: 10.1186/s12905-018-0518-z (PMC5800284; doi:10.1186/s12905-018-0518-z)
Supplement: Additional file 1: — Interview guide. (PDF 240 kb) [file 12905_2018_518_MOESM1_ESM.pdf]

Additional file 1: Interview guide.

Interview 1 – During pregnancy

(Main questions with probing questions to be used if not covered in the main)

1. Tell about when you fell ill/was informed about having gestational diabetes (GD)!

How was it detected? What happened? What did you think? What did you do? Where did you turn for advice/help (firstly/secondly)?
Cause of GD? How long do you think it will persist?
What do you think about your own/the babies health/future health?
Is there anything you are afraid of as concerns GD/the future?
Problems/consequences of GD?

2. What does good glycaemic control mean? How to reach?

Controls? Values?

3. What does health mean to you?

Factors of importance for health for you/the baby? Influence of others/healthcare staff?
What does your economy mean for your health?
Factors negatively influencing? How to maintain health?
Is there any measures (e.g home remedies/use of CAM/prayers/ceremonies/traditions/self-help groups) perceived improving health now when having GD?
Describe what is considered as good/appropriate diet/exercise/self-controls for you as you have GD!
What advice have you got about diet/exercise/medications/self-controls/visits to physician or nurse?
Do you follow them? If not, why?

4. If you need help or advice for health; where do you turn (firstly/secondly)?

Alternative treatment? Influence of religion?
Perceived accessibility in healthcare? Perceived contact with healthcare staff? Do you get help needed? Expectations on staff? Do you follow advice given? If not, why?,
Is there anything you perceive as problematic in contact with healthcare staff controlling you?
How do you think that a good care for a person with GD should be planned/designed?
Is there anything you lack in the care you receive? If yes, what?
How do you think a good nurse/doctor should be?

Interview 2 – 3 months after delivery

(Main questions with probing questions to be used if not covered in the main)

1. Tell about your situation today with a 3 month old child! How do you perceive it?

2. At the previous interview we talked about when you fell ill/became informed about having GD and how you thought and felt then. How do you think about that today when you look back? What do you think about your own/the babies health/future health?

How long do you think GD will persist?
Is there anything you are afraid of as concerns GD/the future?
Problems/consequences of GD?

3. How do you control your GD? How do you live now compared to before being pregnant/during pregnancy?

Follow previous advice? If not, why?
Support from healthcare to be able to have a good health? What support is needed? Lack of something? Problems perceived?

4. What does health mean to you?

Factors of importance for health for you (concerning GD?)/the baby? Influence of others/healthcare staff?
What does your economy mean for your health?
Factors negatively influencing? How to maintain health?
Is there any measures (e.g home remedies/use of CAM/prayers/ceremonies/traditions/self-help groups) perceived improving health now when having GD?
Describe what is considered as good/appropriate diet/exercise/self-controls for you as you have GD!
What advice have you got about diet/exercise/medications/self-controls/visits to physician or nurse?
Do you follow them? If not, why?

5. If you need help or advice for health, where do you turn (firstly/secondly)?

Alternative treatment? Influence of religion?
Perceived accessibility in healthcare? Perceived contact with healthcare staff? Do you get help needed? Expectations on staff? Do you follow advice given? If not, why?,
Is there anything you perceive as problematic in contact with healthcare staff controlling you?
How do you think good care for a recently become mother with GD shall be planned/designed?
Is there anything you lack in the care you receive? If yes, what?
How do you think a good nurse/doctor should be?

Interview 3 – 14 months after delivery

(Main questions with probing questions to be used if not covered in the main)

1. Tell about your situation today with a 14 month old child! How do you perceive it?

How have you perceived care/support from healthcare during the year since we met the last time (3months after delivery)? Has delivery and motherhood implied any problems for you and your health since you have got the diagnosis of GD? If yes, what? Have you got support/help needed? Is something lacking?

2. At the previous interview we talked about when you fell ill/became informed about having GD and how you thought and felt then. How do you think about that today when you look back? What do you think about your own/the babies health/future health?

How long do you think GD will persist?
Is there anything you are afraid of as concerns GD/the future?
Problems/consequences of GD?

3. How do you control your GD? How do you live now compared to before being pregnant/during pregnancy?

Do you follow previous advice? If not, why?
What support do you get from healthcare to be able to have a good health? What support do you need? Do you lack anything? Do you perceive any problems?

4. What does health mean to you?

Factors of importance for health for you (concerning GD?)/the baby? Influence of others/healthcare staff?
What does your economy mean for your health?
Factors negatively influencing? How to maintain health?
Is there any measures (e.g home remedies/use of CAM/prayers/ceremonies/traditions/self-help groups) perceived improving health now when having GD?
Describe what is considered as good/appropriate diet/exercise/self-controls for you as you have GD!
What advice have you got about diet/exercise/medications/self-controls/visits to physician or nurse?
Do you follow them? If not, why?

4. If you need help or advice for health, where do you turn (firstly/secondly)?

Alternative treatment? Influence of religion?
Perceived accessibility in healthcare? Perceived contact with healthcare staff? Do you get help needed? Expectations on staff? Do you follow advice given? If not, why?,
Is there anything you perceive as problematic in contact with healthcare staff controlling you?
How do you think good care for a recently become mother with GD shall be planned/designed?
Is there anything you lack in the care you receive? If yes, what?
How do you think a good nurse/doctor should be?
